# Joint Optimization of Process Flow and Scheduling in Service-Oriented Manufacturing Systems

**DOI:** 10.3390/ma11091559

**Published:** 2018-08-29

**Authors:** Joe Vargas, Roque Calvo

**Affiliations:** ETS Ingeniería y Diseño Industrial, Universidad Politécnica de Madrid, Ronda de Valencia, 3, 28012 Madrid, Spain; joe.vargas.villa@alumnos.upm.es

**Keywords:** priority dispatching rules, simulation optimization, job shop scheduling, flexible manufacturing systems, service-oriented manufacturing systems, maintenance, aircrat engine repair and overhaul (MRO)

## Abstract

Customer-oriented management of manufacturing systems is crucial in service-oriented production and product service systems. This paper develops the selection of dispatching rules in combination with alternative process flow designs and demand mix, for a maintenance, repair and overhaul center (MRO) of turbo shaft engines, both for complete engines and engine modules. After an initial systematic screening of priority dispatching rules, the design of experiments and discrete-event simulation allows a quantitative analysis of the better rules for the alternative process flows with internal and service metrics. Next, the design of experiments with analysis of variance and the Taguchi approach enables a search for the optimal combination of process flow and dispatching rules. The consideration of extra costs for overdue work orders into the costing breakdown provides a quantitative evaluation of the optimum range of load for the facility. This facilitates the discussion of the significant trade-offs of cost, service, and flexibility in the production system and the operational management alternatives for decision-making.

## 1. Introduction

In a global competitive market, service-oriented manufacturing systems follow a path where gaining and retaining the customer becomes fundamental for sustainability. From product service systems (PSS) [1] to classical flexible manufacturing systems [2], passing through service-oriented flexible manufacturing systems to a different extent [3], all of these taxonomies refer to system configurations where classical or new manufacturing competitive factors trade off system performance for service through operational decision-making. Maintenance, repair and overhaul (MRO) highly qualified manufacturing activities are offered with strong competition in the current global economy. MRO systems include the industrial activities of assembly and disassembly, components repair, inspection, as well as replacement or fault correction of an aircraft or its components, in order to preserve the airworthiness conditions and to guarantee aircraft safety operations [4]. MRO companies follow the technical requirements that aeronautical authorities establish [5]. The MRO sector includes the original manufacturer of the equipment (OEM), airlines or aircraft operators, and MRO independent companies [6]. In this market, OEMs are the biggest players through their after-sale services, with 56% share achieved by 2014 and still growing [7].

Fleet maintenance cost of aircraft operators reached USD 61,100 MM in 2014 [8], and it represented from 10% to 15% of the total operational cost of the aircraft operations [9]. A trend of 3.8% annual growth is expected, reaching about USD 90,000 MM by 2024 [8]. Engine MRO represents about 40% of the total. The MRO activities expansion evolves with heavy competence [10], so MRO companies must focus on excelling their operational effectiveness by reducing costs, stocks and job shop times [9], while holding high quality and reliability, at the level of the aeronautical regulations and standards. In a competitive market, operational flexibility and resources utilization is important in accordance with the business goals in MRO [11]. At the MRO facility level, these requirements are highly qualified experienced personnel together with a proper integration of scheduling and provisioning in the supply chain, both upstream (suppliers) and downstream (customer) [5,12]. In this path, different operational techniques and strategies formerly used only in conventional manufacturing organizations are becoming important in MRO production activities. For instance, conventional repair shop layouts have been transformed into cellular systems in order to improve MRO performance [13].

In this context, both the operations practice and its research can benefit from discrete-event simulation of manufacturing system models. The real complexity of the system can hardly be represented through analytical models and it is only partially tackled by the simulation model [14], but it provides an off-the-line test bench to foresee potential trends of performance and to help in decision making or to validate analytical models [15]. Manufacturing system simulation bears the additional advantage of low cost experimentation close to the configuration of real systems [16], and it can help to improve decision making on new manufacturing systems design. Discrete-event simulation techniques have a broad scope [17]. Four different fields of work can be considered: simulation model development, model use, field application, or discrete-event simulation with other simulation techniques. In the manufacturing research area [18], initially three main different areas of work are the design of the manufacturing system, manufacturing operations study, and software development. In addition, the following can be included [19], the programming of maintenance activities, job shop task scheduling, and the research on simulation of meta-models and optimization.

The increasing importance of manufacturing service activities requires a proper combination of production internal operations with customer service goals. Operations quality improvement in a broad sense enables the increase of sales and the reduction of production costs [20]. Beyond the conformism of an adequate performance, current paradigms of improvement look for waste elimination (lean manufacturing). The Taguchi approach to quality combines the classic meaning of conformity with the aim of optimizing system performance [21] by considering that the gap with the optimum is a waste. Moving from the conceptual approach to the real operations in a MRO facility, customer satisfaction can be influenced by repair quality in a highly regulated activity, but also by the lead-time of maintenance and due date compliance. The concurrent analysis of programming, scheduling, system capacity management, and their metrics follow-up is a complex task [22]. Operations management becomes an integrated effort of customer care and of internal process and resources management [23,24]. In consequence, dispatching rules are important decision-making criteria in the daily operation management, with impact on the overall system results and its logistic operating curves [25].

Dispatching rules define job shop scheduling by priority rules based on performance metrics. There are many different designed and studied dispatching rules. A first classification [25] includes four groups: rules based on the processing time, rules based on the due date, combined rules from the former groups and a group including all the rest. Other classifications have been proposed [26], based on the input data to the rules and the priority rule itself, sorting more than 300 existing rules into nine groups. It can be inferred that choosing the proper rule is neither immediate nor easy. It would depend on the operational context, the own production system, and the more valuable performance metric. A multi criteria approach of processing time, work order tardiness, and utilization of resources [27,28,29,30] seems to be suitable for production systems focused on service.

Sequencing can be considered a decentralized process in the decision problem. Specific research in scheduling service-oriented job shops [31] not only considers the mean time to completion of work orders, but also the overdue orders tardiness accumulation. This last study is based on discrete-event simulation on a schematic facility. Even without detailed description of the processes, it shows that when using finite resources at different degrees of utilization, the ordinary need of tracing the serial number of specific parts adds extra complexity to the repair process. This increases the effort of coordination in final assembly, adding extra time and inventories involved in operations. The priority rule “shortest processing time first” was identified as the best option towards reducing mean flow time. Focused on the service metric of overdue work orders, scheduling by “earliest due date first” was more effective. Also, rules based on the complexity of the bill of materials (BOM) to speed assembly, reparation, and disassembly activities seem to be only effective for simple product structures, with difficulties arising of the serial part number matching. A significant simulation based on field data inputs from a capital goods industry [32] can be considered that partially approaches the scheduling constraints of MRO, in particular for spare parts production. It concludes with a better behavior of the “most remaining operation first” rule for spare parts at product level, but the “shortest operation first” rule is better at component level. In the particular field of aircraft engine MRO, this last result suggests that the system could require for schedule optimization different priority rules in the cases of complete engines overhaul maintenance or only modules maintenance.

MRO is a multi-project scheduling problem due to the diversity of tasks that cannot be fully determined a priori at the work order entrance. Studies that revisited the overall problem of multi-project scheduling [33] show the convenience of different priority rules for different local or global objectives.

A more specific recent study of aircraft engine maintenance [34] includes the relevance of the unsteady flow of work orders content in MRO and the main barrier in reaching due dates with origin in parts procurement and part repair. This study is limited to only one process flow for priority rules analysis, based on simulation and design of experiments. The main result suggests the application of the “slack” rule (higher priority in work orders with the lowest difference between the remaining time to completion date and the remaining processing time), in a decentralized application. Real field tests of the rule gave positive results, maintaining system performance while facing a significant load increase.

A recent research contribution seeks an integrated approach combining the study of dispatching rules with parts pooling sourcing for service [35]. In addition, recent research prospects look for an overall optimization of service-oriented production and business management [36]. This study uses a simple example of theoretical sequencing to illustrate the integration of cost penalties of tardiness in the operation decisions. Work planning is shown in two levels: project and scheduling. Better results are reached in project performance giving priority to jobs with small resource workload. The “minimum worst case slack” rule gives the better results at scheduling level. Former research trends in the integration of planning and logistic factors converge with the recent outlooks of a main aircraft manufacturer [37]. It outlines the increasing importance of inventory pooling and the increasing use of maintenance planning tools by the aircraft owner, together with a need of MROs improvement in speed turnaround time, reducing the time in the job shop.

Improving and extending former studies, this paper presents a combined optimization of alternative process flows with a selection of significant priority rules. Different from the former studies on priority rules that are based on fictitious generic processes or a single process as a bench case, this paper approaches the design of the MRO system to real options configuration, facing the problem of mix work orders (overhaul and modules maintenance) in aircraft engine MRO. In addition, the study includes the analysis of overall costing results of service penalties, a real situation only conceptually treated in former studies.

This paper is organized as follows: in Section 2, the process flow of the MRO is analyzed. Section 3 presents the MRO system models development for discrete-event simulation. Next, Section 4 includes the selection of a set of dispatching rules by testing them through simulation, and a scoring process. System behavior and its implications in operational curves are analyzed. Section 5 covers the analysis looking for the best combination of process flow and dispatching rules in relationship with system metrics, with the Taguchi techniques, and cost analysis including charges for overdue work orders, and presents a synthesis of discussion and analysis of the former results with concluding remarks.

## 2. Materials and Methods

### 2.1. MRO Production System: Layout and Process Flow

Turbo shafts are aircraft engines ordinarily used as helicopter power plants. They are characterized by their compact size, low weight, high power, and high reliability [38]. Current aircraft engines, in particular the turbojet engines and their variants, are based on modular design and manufacturing. The modular conception has also implications for operation, fault diagnosis, and maintenance [39]. The typical configuration of a turbo shaft engine includes a cold section module, made up of the compressor; the hot section module, including the combustion chamber, and the high-pressure turbine, which drives the compressor, and finally the power section module that delivers the power to the main shaft.

**Figure 1:**
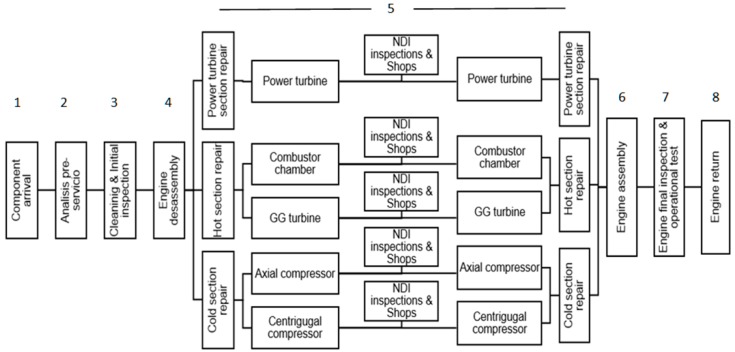
Cell-oriented overhaul process flow.

The more complex and deeper maintenance program is the overhaul. It includes the overall engine inspection and repair. The modular build of the engine allows conduction of the maintenance of every module independently, so personnel, tools and shop layouts are focused on each module. There are eight main chained stages of maintenance: (1) engine shop income; and a (2) pre-analysis that allows a proper planning of the tasks in relationship with the customer’s requirements and engine condition records; (3) initial inspection, with general cleaning that allows a proper visual inspection looking for faults and component damage (corrosion, wear, etc.); (4) engine disassembly, to separate modules from each other; (5) module repair, based on its condition, including cleaning, disassembly sub-sets and detail inspections with proper recovery of condition, followed by re-assembly; (6) engine assembly from modules; (7) bench test; and (8) engine conditioning and storing before the delivery to the customer. These steps can be more clearly identified in the Figure 1 process chart.

Across the different overhaul steps, quality assurance includes an airworthiness guarantee of engine components. The physical layout of the plant is an important productive factor of flexibility, response capability and finally competitiveness of the MRO organization. The shop includes reception and shipping areas, assembly/disassembly zones, special inspections (non-destructive inspection and testing) area, and in-house repairs of different techniques.

The alternatives of production organization can be oriented to the process or to the product [40]. These orientations influence production plant layouts, so production lines follow the sequence of the processes, and production cells typically support families of products (engine modules in this case).

Production with layout process-oriented is in relationship with a high volume and low variety of the product. The division and specialization of labor and a proper line of balancing provides high labor utilization. In addition, a proper design of capacity for a stable demand is required to reach high efficiency. In the extreme case, a production with forecasted demand without uncertainty could be optimized in dedicated lines. In the case of modular aircraft engines of a particular model or family, the maintenance and production can be planned in a sequence where every module is refurbished in every work center of the line. Because the necessary repair operations at each stage are not the same for every engine, it appears the unavoidable imbalance in queues at each work center of the production line, with the result of longer lead times.

In the case of production layout and product-oriented planning, human and material resources are organized by thinking of the variety of products and customer needs. Instead of the same rigid sequence of operations of the production line, production systems organized by product can allocate a great variety of different sequences (routing across the layout) depending on the product configuration. Nevertheless, a general big drawback could be a lower utilization of resources. This type of production system configuration can be applied in engine overhaul to every engine module, so its maintenance is planned and executed independently.

Another current main type of production system is the manufacturing cell. It is oriented to product, but taking into account the process commonalities of the different products of a family. This means that the families share processes, more than the product functionality. The organization around production cells requires the identification of those commonalities across the product portfolio, placing high skilled workers and transforming the layout design around the cells. Efforts to reduce waste in all forms guide their configuration. This organization provides good results in throughput, productivity, and quality [41]. Inspired in the principles of waste reduction of lean manufacturing, MRO facilities have initiated the adoption of this approach, but less generalized than other ordinary manufacturing systems. That is because MRO is frequently focused on customer satisfaction over the product or process constraints [13].

In the case of aircraft engines, its modular architecture gives commonality in the operations of maintenance across engine models. That is, for instance, the maintenance tasks for the hot section of different engines share similar processes. A process of engine overhaul under such premises is depicted in Figure 1. It contains cells by the two different types of compressors, the combustion chamber and the two turbines. This configuration in cells requires extra working shop area and investment, but it obtains some independence and specialization in the processes, from which a shortening of production time with better service is expected. This will be the basis of the production layout considered hereafter for study.

The layout determines the routing of the engine or its components across the plant. For every engine, its modules or part of them (just components in general), can be repaired by routing them to every step (disassembly, inspection non-destructive testing, repair, and assembly) and after task completion they wait in a meeting area until the three modules have been finished at that step, in order to pass to the next one. This flow process guarantees the engine (work order) evolves through the facility together as a whole entity, with easier flow control of work orders. In the case of work orders of modules, they do not wait, so they pass through the system without visiting the waiting area. For complete engines or modules, we call this process flow synchronous, model S.

Alternatively, the components can be routed to the proper repair shop independently and once the tasks have been finished, they can wait in a meeting area downstream to the rest of the components, just prior to final assembly. We call this asynchronous flow, model A.

### 2.2. MRO Model and the Discrete-Event Simulation of the System

The base model of Figure 1 under two workflows, synchronous S and asynchronous A, is considered for discrete-event simulation, using ARENA software, Rockwell Automation. In the simulation model, the components (modules or part of them, also named modules for simplicity) are entities of four types: motor AA (full engine including its three modules), module TP (power turbine), module SC (hot section), and module SF (cold section). The entity attribute declares the properties to route or make decisions about every module. The following attributes are associated with each entity (work order): STEP (routing step), NSTEP (locate at meeting area), PRODUCT (product identification at each step entrance), TPROCESS (time of process at every process step), TINSPECTION (inspection time at non-destructive testing NDT), PRIORITY (priority assignation at the queue).

#### 2.2.1. Maintenance Sequence

The sequences defined in the model are used to establish automatically the order of process execution in inspection and repair and to establish the processing times and priorities, Table 1 and Table 2. The assignation is done by accounting for every entity and its status, at every step of the maintenance process.

#### 2.2.2. Production Process, Process Time, and Resources

One of the advantages of discrete-event simulation is an easy modelling of process duration variability and the events occurring by probabilities distributions [42].

In order to choose the better probability distribution to fit the variability of the process times or events occurrence, the proper behavior of the modelled feature is essential. Some functions are used to approach real behavior: triangular distribution for the process time, Table 2, at each workstation or exponential distribution to simulate the arrival of entities and uniform distribution to assign the type of component.

Processes and resources are both companions in the modelling process. Every process is accomplished by its machinery and/or human resource assignations. The simulation framework computes their use associated with the task so the utilization can be calculated. The duration of the tasks together with the limited number of resources assigned to them, both bound the capability of the workstations to tackle every work arrival, so the queues couple workflow and workstation capabilities. Different types of specialized human resources are assigned to the different processes including technicians for general tasks, specialists in different processes, and propulsion engineers like supervisors and/or decision-making [43].

#### 2.2.3. Simulation Runs

In addition to the setup of the simulation model described above, a proper simulation requires the minimization of bias in the results by the effect of initial conditions. The initial behavior of a system awaiting and empty is not the stable state that is sought through simulations studies. The method adopted to determine a stabilization time [44] consists of two steps. First, the number of run replicates necessary to reach the half-width of desired maximum variability, by (1). Where *η* is the number of necessary replicates, *η*_0_ the number of initial replicates (set to 10), *h*_0_ the initial half-width reached, *h* the desired half-width. Next, graphically the point is established where the transient period finishes.

(1) $$\eta =\eta_{0}\cdot h_{0}{}^{2}/h^{2}$$

**Figure 2:**
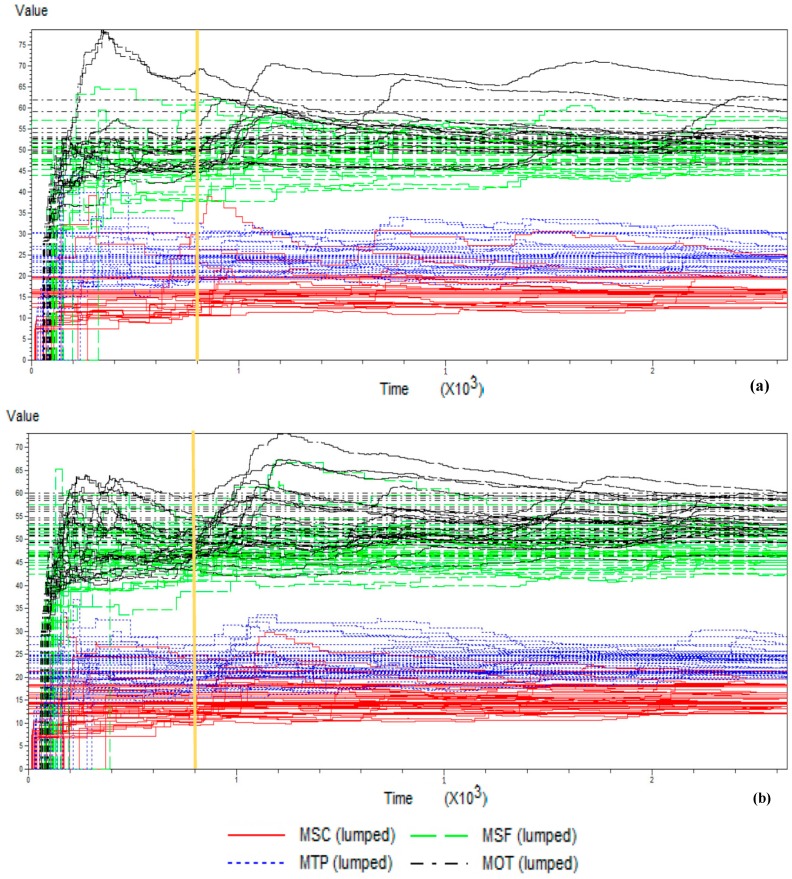
Simulation warm-up period estimation: (**a**) For model S, (**b**) for model A.

The calculations show that at least 22 replicate runs for model S and 15 for model A are necessary to come up with the desired half-range in the worst case. The next step requires fixing the overall conditions of the simulation. The particular setup conditions include: 22 warm up replicates for model S and 15 for A, 400 days for stabilization plus 1825 days of simulation run, an average of eight units per month with random demand under exponential distribution, seasonality with high demand in winter and low demand in summer following a cosine curve. Labor days of 16 h are used with continuous availability of resources, and neglecting at first approach the transportation times inside the facility. The full model programmed in Arena is run to determine the overall behavior, as in Figure 2 [43], in order to estimate the stabilization period of the different parameters across the baseline time.

## 3. Results

### 3.1. Integrated Analysis of Process Flow and Dispatching Rule

Operations management in an MRO facility holds two main global objectives: customer service and internal performance. Customer service is defined as the proper quality level, including the technical proficiency that complies with regulations and achieves the agreed short lead times at a competitive cost. The internal objective requires operations with a proper resource assignation, so that the full capacity of the facility should be used effectively under variable demand. The implications of the operations under uncertainty involves trade-offs of service ratios and manufacturing flexibility [45], including the external position (market share) and internal performance (maximum capacity compatible with the intended service ratios and proper resources utilization at partial load).

Dispatching rules are tools for operations management and decision-making. In the customer service field, this includes reaching the due dates to avoid extra costs for delay, but taking into account the complex relationship of multiple work orders in processes of different customers, with different task times, number of operations or engine delivery due date.

In the MRO operational context, choosing the more convenient priority rule is not at all evident. In a first screening process, eight noteworthy dispatching rules from the literature are studied, Table 3. They are selected based on the objective, but also on the practical applicability. The simulation model of the MRO facility is run under these different rules and for different demand profiles.

The first five rules have the main objective of reducing the delay time and the number of delayed deliveries, which is to say, coping with customer service. They include operation parameters like due date, processing times, as well as the total process time or remaining operations at each step. The rules largest processing time (LPT) and shortest processing time (SPT) take global consideration of the work order content. The rule LPT prioritizes work orders of engines under overhaul, instead of engine modules, taking care of the higher costs to the customer due to the engine out-of-service cost (including leasing or insurance charges). Meanwhile SPT gives precedence to work orders of engine modules before the overhaul of engines. Engine modules require less repair time and the delivery times are shorter. Finally, the FIFO (first in, first out) rule is an initial reference baseline for comparison purposes. 

Considering the setup of the simulation model of Section 3, the results are included in Table 4. The average values allow a comparison of synchronous (S) versus asynchronous (A) models. It can evaluate the general limitations of the facility model in a pair wise comparison across priority rules. The model A behaves slightly better in the metrics associated with work orders and time. Model A performs better than model S for work orders accomplishment, about 8% in wait time, reduces 11% due work orders and 3.6% in work in process (WIP). In relationship with time metrics, Model A improves 4.8% value added and a 4.5% in total lead-time, while utilization is similar for both A and S flow processes.

This result brings a first consideration that in a broad approach across priority rules and with the portfolio of engine modules and overhauled engines, the asynchronous model A performs better than S. It improves service ratios in work orders completion and time metrics. The A flow process is more appropriate in terms of service, speeding up work orders better across the system, and in an exchange with the reduction of inventories (work in process), with similar dedicated resources. That is, the service is improved by reducing intermediate stocks with the same resources over the period. The overall capacity remains practically the same (see the number of complete work orders and utilization).

The relative performance of every priority rule is different for every different metric. The selection of the most appropriate priority rule is a multi-criteria decision-making problem. In order to compare the relative performance between them, the simple well-known FIFO rule can be a reference level, frequently used in queues studies and stock management. Note that the rules LPT and SPT do not reach a simulation solution under the limiting condition of a maximum number of work orders in process, so they are left aside initially from any further investigation. Counting the number of performance criteria that are over those obtained by the FIFO rule allows a simple initial ranking of priority rules that outperforms FIFO. The score weights equally the work orders (WO) throughput, average cycle time, and the performance across customer WO. As a result, the rules CR+SPT, EDD, and SPT (this one only for the synchronous flow S) obtain total positive scores, outstanding over the FIFO reference. In addition, the CR rule performs better for overhauled engines than FIFO. Therefore, the rules CR+SPT, EDD, and CR (for full engine overhaul) are selected for further study by a design of experiments.

### 3.2. Logistic Operating Curves

Even when in the case of MRO its service metrics play the main role in decision making, conventional internal manufacturing trade-offs can be surveyed by the logistic operational curves [25]. They are put into relationship output with the product level inventory (work orders in process). Note that the spare parts inventory and its availability is another face of the whole MRO operational scenario, not tackled directly in this study. For given resources, a priori operation with high output and low level of inventory contributes to internal efficiency and service.

**Figure 3:**
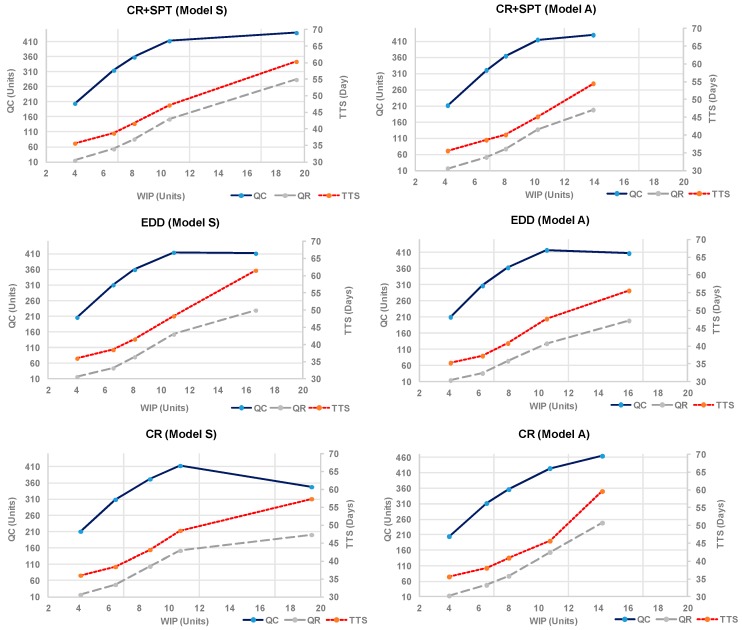
Logistic operating curves under selected priority rules and process flow models.

We can consider the slope value of the operating curves as a sign of performance per unit of inventory (WIP) for the completed work orders (QC). Conversely, the quantity of late work orders (QR) and the lead-time in the system (TTS) are sought to be reduced, so the lower the slope of the curve, the better performance with respect to inventories. These operating curves, Figure 3, show the system performance for a demand mix with 70% engines for overhaul.

The results show that the synchronous flow S performs slightly better under the rule CR+SPT for the maximum total output (QC). In terms of lead time (TTS) and late work orders (QR), the rule CR+SPT works also better over 10 units of WIP, and all three rules have a similar behavior under that level.

Under the asynchronous flow A, the system reaches the higher output (QC) managed by the CR rule, but at the cost of worse (TTS) and (QR) metrics, while the best is EDD.

Comparing the results between models, the output the asynchronous model A reaches higher output ruled by CR, followed by CR+SPT. In general, the operating curves show higher slopes under 10 units of inventory than over the 10 units. That level of work-in-process can be considered an operative reference level convenient for the size of the system under study. Under that level, the growth of inventory serves to increase output, but after that level only marginal increases of output are obtained, and always at the cost of increasing lead-time in the system (TTS) and late work orders (QR).

## 4. Analysis and Discussion

### 4.1. Operations Variability and Evaluation through the Taguchi Approach

Even when the simulation model includes variability in the internal conditions of operations of processing times, the variability of controlled and not controlled factors can be studied in a more systematic manner through the design of experiments (DOE) and the Taguchi approach. The goal of this analysis is to find the more robust internal control factors opposed to the external uncontrolled factors that cause variability. Operational metrics are the numbers of late work orders, average total time of work orders in the system, inventories (work-in-process), and the relative cost evaluated through the utilization of resources. In particular, for the experiments, the work order mix (percentage of overhauled engines versus engines modules) and the priority rule are controllable factors, Table 5. Meanwhile, the demand level, the learning or progress curve and the percentage of rework in the shops are variability factors included as noise factors bounded in each simulation.

The response variables correspond to the operational metrics that are sought to be robust, Table 6. This represents the optimization of its value (maximum or minimum objective) versus its variability (noise); that is, the Taguchi approach of maximizing the signal-to-noise ratio. This DOE considers the analysis of four different response variables: Total time in the system (TT), late or delayed works (DW), relative cost from the utilization of resources (BRC), and in process inventories (work-in-process, WIP).

The criteria of the smaller-the-better minimizes the mean and the standard deviation of the response when maximizing the signal-to-noise ratio [46]. The case of larger-the-better criteria, maximizes the mean and minimizes the standard deviation. Finally, the criteria nominal-the-best minimizes the standard deviation or variation. The experimental setup and array are also detailed in Table 6. It includes internal and external orthogonal arrays for the controlled and noise factors, and the number of levels that allow discrimination of the influences.

The results of the experiments through simulation are summarized in Table 7. The column signal-to-noise ratio shows the results of the best level of the controllable factors (LEV) together with the contribution to the variability (VAR) of the mix and the priority rule. The contribution result is included only when the variability contribution of the priority rule is significant. In the column signal-to-noise the optimum levels show the results through the criteria of smaller-is-better or larger-is-better, as per Table 6. As labelled, in the column signal-to-noise ratio nominal the best, the optimization criterion minimizes only the response variability. Finally, the column mean response gives the results for the sensitivity of the response parameters.

The synchronous (S) process flow model exhibits a better general behavior for the mix of 60, because is the best level for TT, DW, and WIP. Note that the criteria of larger-the-better for the relative utilization in the synchronous model require a facility working with 80% work orders for overhaul engines; an exigent situation from the point of view of customer’s service management. Simultaneously the criteria nominal-the-better presents the best response (minimum variability) for the 60% mix in combination with the CR+SPT priority rule for three of the criteria, but DW is always better for EDD. In overall terms, the synchronous flow process (S) presents the best response for a middle mix (60% overhaul engines and 40% engine modules) in service metrics (TT, DW), and also WIP. In addition, this mix is favorable for internal facility management because it corresponds to the better level for internal inventories (WIP) and minimizes the variation of relative cost of utilization BCR of internal resources (nominal-the-best) that is a convenient departing point for ulterior internal improvement actions. Focused exclusively on customer service response and in particular on the potential customer extra costs for late work orders, the rule EDD seems to be more convenient in the synchronous flow process together with the mix level 60. The rule has a remarkable influence (up to 40%) in the variability of the mean response of due or delayed work-orders (DW).

The asynchronous flow process A behaves in a similar qualitative overall way, presenting better customer-focused response in service by working at level of 60. The mix level 60 is better for the response metrics TT, DW, and WIP. In addition, onlyt focusing on the costs of facility operations, the higher the level of overhaul engines resulting, the better the performance. Although, the dominating rule for DW optimization is in this case CR+SPT instead of EDD.

The results of the DOE are compatible and complementary with those obtained in the logistic operating curves. In these curves, the mix under consideration was at the intermediate level of the experiments, 70% engines for overhaul, in between 60% and 80%. The operational curves show the mean results of simulations, but with a fixed setup. Conversely, the experiments evaluate mainly the variability behavior with alternative setups, more interesting for the design of the system than for its operations. Both approaches complement each other for system design and operation.

The results of the DOE show little influence of the priority rule when the percentage of full engines for overhaul increases in the mix. In accordance with the analysis of variance, the optimum results at the level of mix 80% (utilization ratio, BCR) are practically determined by the mix itself and with very little influence from the priority rule.

Both models’ results, S and A, show reasonable trade-off trends: the optimization of cost in the system can be associated with a higher utilization of available resources through full service (overhaul engines versus modules repair, in an MRO facility). Nevertheless, service to the customer is associated with the service ratios DW and TT. Therefore, flexibility for a better response requires the non-full utilization of internal resources, coping with a mix that contains not only engines, but also modules. That is, for a good customer response, flexibility maintains a trade-off with the internal resources utilization. The energetic behavior of internal efficiency—the greater the amount of full service work orders, the better the utilization that results—seems to be out of the path of service metrics (DW and TT) and even of the general principles of current facilities management for inventories minimization (WIP).

These results are consistent with previous studies, and they expand former research. Focused only on service, the dominance of the rule EDD (earliest due date) first for the synchronous model S is also in the findings of Guide et al., 2000, even when their research is based on a generic non-detailed process. In the same sense, a recent contribution based on just one process flow [34] gives the better result in terms of service using the slack rule. This is the case for the asynchronous model A in the present study. Note that the combined rule CR+SPT is a hybrid rule of two factors that included the slack rule rated by the remaining processing time (see Table 3). Since both former studies used different process designs, the difference between them has not been previously discussed nor their differences interpreted, so the importance of the coupling of process flow with a priority rule remained hidden, conversely to the findings of the present study.

### 4.2. Cost Sensitivity Analysis

The general results show a better behavior of the system in terms of service when a significant participation of modules in the work orders exists. Conversely, the optimization in terms of cost for proper resources utilization, BCR, asks for more work orders of engine overhaul. In general, scheduling a service-oriented manufacturing system must pursue customer satisfaction [47]. Due to the high value of the availability of aircraft engines, the costs of the operation of an MRO cannot disregard the contractual charges due to delays or overdue work orders. Airlines exploit aircraft engines through flight operations, so each day the aircraft is on the ground due to maintenance impacts with revenues losses [48]. The MRO activity shares these impacts, so charges are agreed for overdue work orders. The MRO manufacturing service facility must consider them in the operative analysis for risk management and decision-making.

A cost of 20% contribution of direct overheads in the total cost breakdown is considered. Its quantification is taken directly from the simulation results. Other direct costs are the materials and subcontracted repairs that can be up to 70% and 10% of the direct cost, respectively [6]. Indirect costs associated with the MRO facility include power, consumables, general service maintenance or real state expenses. Those costs can be up to 40% of the total cost of MRO operations [2].

The charges of delayed work orders should be a function of the value of the hardware and the amount of the delay. Rates of 20,000 EUR per day for full engines and 15,000 EUR per day of delivery delay in the case of modules are considered [49]. In fact, those penalty charges are only a small portion of the real operational cost impact to the customer. The repercussion on customer loyalty and business reputation might go beyond those figures for the MRO facility.

The cost study is setup with a range of demand from 4 to 10 work orders per month. Profiting from the best results from the DOE, the model S is run under the rule EDD with a 60% of overhaul. In the case of model A, the dispatching rule is CR+SPT, and with a 60% of engine overhaul.

**Figure 4:**
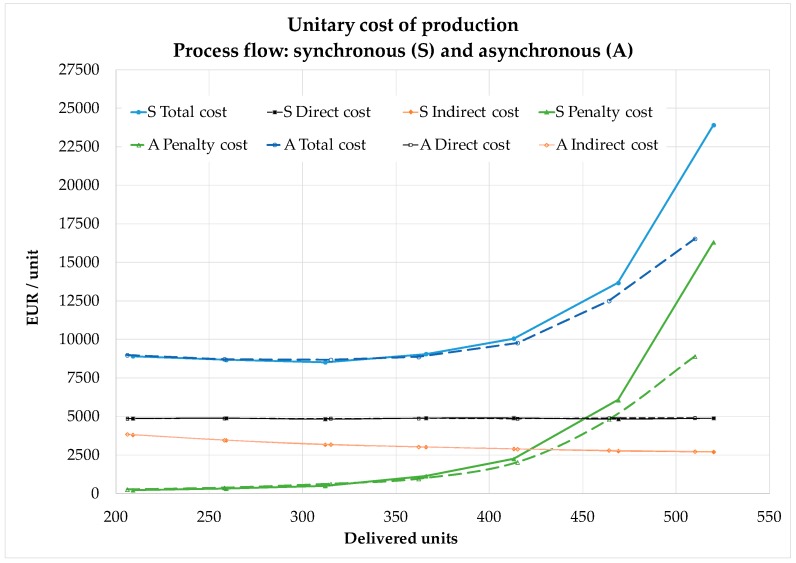
Unitary cost analysis breakdown in a 5 year simulation period.

The results of the simulations are represented in Figure 4. In the *x*-axis the total work orders or units processed are presented. The *y*-axis shows the different components of unitary cost. The total cost is composed of direct and indirect components plus the variable cost with origin in the overdue work orders charges. This extra cost impacts at high load level and establishes an optimal minimum cost in the range between 300 to 350 work orders in a simulation horizon of 5 years, or an equivalent average annual demand from 60 to 70 work orders for the system under analysis. In the comparison the behavior of both process flow models, the asynchronous flow A responds slightly better to high load, in terms of the expected extra charges for delays. The better behavior of the process flow A together with the compound slack rule (CR+SPT) is consistent with the better results found in recent research [36] where the penalty in MRO operations is explored based on a simple generic process.

The situation of overload might not be an ordinary state and it can be considered to establish facility design boundaries or expansion flexibility [45]. Focusing on the ordinary range of operations, the total unitary cost is similar for both process flows. The convenient range of lower cost per delivered unit is from 60 to 70 annual work orders. In this range, the extra costs per overdue work orders could be around 8% to 12% of the total cost, but operating at around 80 work orders per year can bear about 25% of extra cost. In addition, note that working at middle load with 40 work orders per year, with charges only representing 3% of total cost, is a range where the underutilization of resources dominates the unitary cost, due to the low load of the system.

## 5. Conclusions

System simulation and the design of experiments allow the quantitative evaluation of the significant effects in operations and the convenient dispatching rules for each process flow. System operating curves can be estimated, so the throughput and inventory trade-offs can be assessed. The influence of work in process inventories in service ratio and in system flexibility are important reasons for paying attention to the adequate level of inventories in the overall performance in service-oriented systems. In addition, simulation allows the evaluation of the convenient range of work load into the system.

The work order mix of the demand (engines and modules) is demonstrated to be an important influence factor to obtain good operational results. The optimization of the resource utilization leads to full capacity use through work orders of engine overhaul, but this situation does not represent the better scenario for service metrics. Being conscious that demand mix cannot be fully controlled, customer service managers might conduct customer portfolio for the more convenient mix combination of engine overhaul and modules through demand management. For the detailed model of the MRO facility under study, the dispatching rules analysis results show a better performance for Earliest Due Date (EDD) rule and Critical Ratio and Shortest Processing Time (CR+SPT) rule in a system with a mix of engine overhaul (60% in the system under study). When each flow is managed with the proper dispatching rule, there is no conclusive advantage in performance based on synchronous and asynchronous flow. Synchronous flows appears to be better run through the EDD rule, while the asynchronous flow better by the the CR+SPT rule. Considering the current maintenance trend to improve life cycle management and pooling of components, the asynchronous flow with the (CR+SPT) rule can be an advantage, approaching better the pooling operation of components.

In service oriented manufacturing systems, supplier’s metrics include quality levels and service ratios, and overdue work orders can generate charges to the supplier. Including the charges of overdue work orders can become significant for MRO centers operational assessment. The quantitative results show that with an increase of overdue work orders extra costs would balance the benefits of increasing load by the reduction of internal cost of the better resource utilization. Other additional effects of overdue orders are also possible, such as the position in the market or customer loyalty. The methodology has allowed the assessment of the convenient range of load that minimizes the unitary cost. In the system under study, when surpassing that range the extra costs of charges increase rapidly. Below that range, operations can benefit from operational flexibility with a stable unitary cost over a wide interval of load. Considering the two process flow type alternatives, slightly better behavior has been shown with high load by the asynchronous flow process in the operating curves and the unitary cost. When considering mainly the service performance and its variability, the synchronous model has shown general more robust behavior in the simulation experiments. Beyond the absolute quantitative differences, it is important that the selection of the proper priority dispatching rule associated with the flow process, be in accordance with the relative management importance given to each metric, in a complex multi criteria decision-making process.

The combined methodology of discrete-event simulation and design of experiments (DOE) through the Taguchi approach allows the detailed consideration of the main influential factors in system operations in relationship to a particular process flow. The operational decision-making improves under the complex relationships between system design and operations management (priority or dispatching rules), looking for the optimal priority rules for a particular manufacturing system. With the same decision-making metrics, the optimal dispatching rule appears to be associated with a particular process flow. In the particular case of the engine MRO center under analysis, many processes and tasks are established by the airworthiness standards, so the system process flow options can be designed by taking into account an integrated analysis of the internal and service ratios. Discrete-event simulation combined with the design of experiments is a convenient technique to evaluate scalable models of manufacturing systems focused on service, including their design alternatives. In general, the trade-off between productive factors and service ratios recommends to look for a process flow design and dispatching rules with favorable results in cost, but in tight relationship with the operational competitive advantages of flexibility and a proper service. 

Former results suggest that future research works can benefit from discrete-event simulation, in addition to other method use (constraint programming, meta-heuristics, agents, etc.). They should combine the analysis of process flow and priority rules with the flexibility under partial resource utilization, in order to cope with load fluctuations while reaching the desired service. In direct connection with the results, the current trend in life cycle management of capital goods (e.g., aircraft engines) encourages pooling systems of equipment or components. Therefore, the study of external coupling of MRO scheduling with hardware management might be probably better studied through asynchronous process flow.

## Figures and Tables

**Table 1:** Sequences of inspection and repair for each entity.

Command	Function	Sequence	
Sec Mod TP	Inspection route for module TP	dye penetrant inspect—waiting area ^(1)^—repair zone	
Sec Mod SC	Inspection route for module SC	eddy current inspect—waiting area ^(1)^—repair zone	
Sec Mod SF	Inspection route for module SF	magnetic particles inspect—waiting area ^(1)^—repair zone	
Command	Function	Sequence	Assignation
MEC1	Special repair route assignation	machining—welding—machining—heat treatment—bonding—waiting area ^(1)^—assembly area	Mod. TP: 36% Mod. SC: 15% Mod. SF: 46%
MEC2	Special repair route assignation	machining—welding—machining—heat treatment—bonding—waiting area ^(1)^—assembly area	Mod. TP: 30% Mod. SC: 0% Mod. SF: 15%
SOL1	Special repair route assignation	machining—welding—machining—heat treatment—bonding—waiting area ^(1)^—assembly area	Mod. TP: 14% Mod SC: 25% Mod. SF: 16%
SOL2	Special repair route assignation	welding—machining—bonding—waiting area ^(1)^—assembly area	Mod. TP: 9% Mod. SC: 20% Mod. SF: 12%
REC1	Special repair route assignation	bonding—waiting area ^(1)^—assembly area	Mod. TP: 11% Mod. SC: 40% Mod. SF: 11%
Engine assy	Assembly engine route assignation	waiting area—assembly area	

^(1)^ Applicable only to simulation in model A.

**Table 2:** Process times.

PROCESS TIME [man hour]	Min	Mean	Max	PROCESS TIME [man hour]	Min	Mean	Max
Initial inspection	10.6	12	14.3	Welding repair (SC)	6.1	7	8.3
Disassembly/Inspection (TP) module	22	26	29.8	Welding repair (SF)	19.3	23	26.1
Disassembly/Inspection (SC) module	20	24	27	Heat treatment (TP)	8.4	10	11.4
Disassembly/Inspection (SF) module	40.7	48	55.1	Heat treatment (SC)	4.9	6	6.6
Engine disassembly	14.9	18	20.1	Heat treatment (SF)	13.8	16	18.6
Eddy current inspection (SC)	3.6	4	4.8	Bonding repair (TP)	10.1	12	13.7
Magnetic particles inspection (SF)	30.1	35	40.7	Bonding repair (SC)	9.8	12	13.3
Dye penetrant inspection (TP)	25.6	30	34.6	Bonding repair (SF)	33	39	44.7
Dye penetrant inspection (SC)	4.2	5	5.6	Assembly TP	51.8	61	70
Dye penetrant inspection (SF)	35.6	42	48.1	Assembly SC	37.1	44	50.2
General repair (TP) module	85.6	101	115.9	Assembly SF	136.4	160	184.5
General repair (SC) module	24.5	29	33.2	Balancing (TP)	13.1	15	17.7
General repair (SF) module	283.1	333	383.1	Balancing (SC)	9.5	11	12.9
Machining repair (TP)	13.1	15	17.8	Balancing (SF)	8.9	11	12.1
Machining repair (SC)	3.7	4	5	Engine assembly	22.3	26	30.2
Machining repair (SF)	55.3	65	74.8	Inspection and final test	10.7	13	14.5
Welding repair (TP)	5.1	6	6.8	Delivery to customer	2.6	3	3.5
Engine assembly total [man hour]		1265.17					
TP module total [man hour]		292.68					
SC module total [man hour]		160.64					
SF module total [man hour]		787.42					

**Table 3:** Selected priority rules.

Rule Name	Formulation
Critical Ratio and Shortest Processing Time (CR+SPT) [27]	$Z=\mathrm{processing}\;\mathrm{time}\times \max\left\{\frac{\mathrm{due}\;\mathrm{date}-\mathrm{current}\;\mathrm{date}}{\mathrm{remaining}\;\mathrm{processing}\;\mathrm{time}},1\right\}$
Critical Ratio (CR)	$Z=\frac{\mathrm{due}\;\mathrm{date}-\mathrm{current}\;\mathrm{date}}{\mathrm{remaining}\;\mathrm{processing}\;\mathrm{time}}$
Slack Time Remaining (STR)	Z=due date-current date-remaining processing time
Slack Time Remaining/Operation (STR/OP)	$Z=\frac{\mathrm{due}\;\mathrm{date}-\mathrm{current}\;\mathrm{date}-\mathrm{remaining}\;\mathrm{processing}\;\mathrm{time}}{\mathrm{remaining}\;\mathrm{operations}}$
Earliest Due Date (EDD)	Z=due date-current date
First In, First Out (FIFO)	Ordering according to entrance time to the system
Largest Processing Time (LPT)	Ordering according to processing time
Shortest Processing Time (SPT)	Ordering according to processing time

**Table 4:** Overall performance under selected priority rules: work orders and time.

**Priority Rule**		**# Completed Works**		**# Due Works**		**% Late Works**		**Work in Progress (WIP) (units)**		
		S	A	S	A	S	A	S	A	
CR		413	425	152	153	36.8	36.0	10.7	10.8	
CR+SPT		413	414	152	136	36.8	32.9	10.6	10.2	
STR/OP		412	410	163	143	39.6	34.9	11.1	10.4	
STR		421	415	169	150	40.1	36.1	11.2	11.0	
EDD		415	417	153	128	36.9	30.7	10.9	10.5	
LPT		421	NA	186	NA	36.9	NA	12.7	NA	
SPT		415	NA	151	NA	36.9	NA	10.5	NA	
FIFO		412	414	159	148	36.9	35.7	10.7	10.4	
average		415.3	415.8	160.6	143.0	37.6	34.4	11.0	10.6	
NA: Due to Arena software restrictions, it was not possible to take data										
**Priority Rule**	**Value Added (days)**		**Wait Time (days)**		**Lead Time (days)**		**Value Added Time (%)**		**Utilization (%)**	
	S	A	S	A	S	A	[%]	A	S	A
CR	48.3	48.3	71.5	66.0	48.2	46.5	40.3	42.2	67.4	69.1
CR+SPT	48.2	48.3	67.6	62.6	46.8	44.9	41.6	43.5	67.9	68.1
STR/OP	48.2	48.2	74.1	67.2	49.3	46.6	39.4	41.8	67.8	67.4
STR	48.3	48.3	71.9	71.5	48.9	48.5	40.2	40.3	68.4	68.2
EDD	48.2	48.2	72.8	68.6	48.1	46.5	39.8	41.3	67.5	68.1
LPT	48.1	NA	87.6	NA	54.8	NA	35.5	NA	68.6	NA
SPT	48.3	NA	66.7	NA	46.3	NA	42.0	NA	68.1	NA
FIFO	48.3	48.3	69.5	65.9	47.6	46.6	41.0	42.3	67.6	67.5
average	48.2	48.2	72.7	66.9	48.8	46.6	40.0	41.9	67.9	68.1
NA: solution not reached under setup constraints due to inventory overflow										
S = synchronous model; A = asynchronous model										

**Table 5:** Design of experiments (DOE) factors, levels and ranges.

Factor	Type	Range	Number of Levels (#)
Dispatching rule (A)	Controllable	CR-EDD-CR+SPT	CR (1)-EDD (2)-CR+SPT (3)
Type of component (B)	Controllable	Engines: 0–100%	60 (1)–70 (2)–80 (3)
Monthly demand (C)	Noise	6–10 units	6 (1)–8 (2)
Learning curve (D)	Noise	95%–85%	95 (1)–85 (2)
Rework (E)	Noise	5%–15%	5 (1)–15 (2)

**Table 6:** DOE response variables and set-up.

Response Variable	Magnitude		Objective	Maximizing		
Total Time (TT)	days		Smaller-the-better	$S/N\left[db\right]=10\cdot Log\left[\frac{1}{\frac{1}{n}\sum_{i=1}^{n}TT_{i}^{2}}\right]=10\cdot Log\left[\frac{1}{TT^{2}+\sigma_{n-1}^{2}}\right]$		
Due Works (DW)	work orders		Smaller-the-better	$S/N\left[db\right]=10\cdot Log\left[\frac{1}{\frac{1}{n}\sum_{i=1}^{n}DW_{i}^{2}}\right]=10\cdot Log\left[\frac{1}{DW^{2}+\sigma_{n-1}^{2}}\right]$		
% Busy resource cost (BRC)	$\mathrm{BRC}\%=\frac{\mathrm{Busyresourcecost}}{\mathrm{Total}\;\mathrm{resourcecost}}$		Larger-the-better	$S/N\left[db\right]=10\cdot Log\left[\frac{1}{\frac{1}{n}\sum_{i=1}^{n}BRC_{i}^{2}}\right]=-10\cdot Log\left[\frac{1}{n}\overset{n}{\underset{i=1}{\sum }}\frac{1}{BRC_{i}^{2}}\right]$		
Work-in-process (WIP)	work orders in process		Smaller-the-better	$S/N\left[db\right]=10\cdot Log\left[\frac{1}{\frac{1}{n}\sum_{i=1}^{n}WIP_{i}^{2}}\right]=10\cdot Log\left[\frac{1}{WIP^{2}+\sigma_{n-1}^{2}}\right]$		
**L_9_ (3^2^) Inner Array**			**L_4_ (2^3^) Outer Array**			
Treatments	Controllable factors		Treatments	Noise factors		
	A	B		C	D	E
1	1	1	1	1	1	1
2	1	2	2	1	2	2
3	1	3	3	2	1	2
4	2	1	4	2	2	1
5	2	2	**DEGREES OF FREEDOM (DOF)**			
6	2	3				
7	3	1	factor	Quantity	Level	Number of DOF
8	3	2	Controllable	2	3	2 × (3 − 1) = 4
9	3	3	Noise	3	2	2 × (2 − 1) = 2

**Table 7:** DOE and ANOVA analysis of results.

Process Flow Model	Resp.	Signal-to-Noise Ratio				Mean Response				Signal-to-Noise Ratio Nominal the Best			
		Mix		Rule		Mix		Rule		Mix		Rule	
		LEV	VAR	LEV	VAR	LEV	VAR	LEV	VAR	LEV	VAR	LEV	VAR
S	TT	60	90			60	90			60	80	CR+SPT	10
	DW	60	60	EDD	30	60	50	EDD	40	60	75	EDD	20
	BRC	80	99			80	99			60	85	CR+SPT	10
	WIP	60	90			60	90			60	80	CR+SPT	10
A	TT	60	90			60	90			60	70		
	DW	60	70	CR+SPT	25	60	65	EDD	30	60	80	CR+SPT	15
	BRC	80	99			80	99			60	75		
	WIP	60	99			60	90			60	70		

Process flow model: S-synchronous; A-asynchronous; Mix: % of full engines in the work orders mix; LEV: experiment setup level; VAR: % contribution to variance; Resp.: response variable; TT: total time in the system; DW: due work orders; BRC: busy resource cost ratio; WIP: work-in-process.
